# Acute Myocardial Infarction in COVID-19 Patients—A Review of Literature Data and Two-Case Report Series

**DOI:** 10.3390/jcm13102936

**Published:** 2024-05-16

**Authors:** Luiza Nechita, Elena Niculet, Liliana Baroiu, Alexia Anastasia Stefania Balta, Aurel Nechita, Doina Carina Voinescu, Corina Manole, Camelia Busila, Mihaela Debita, Alin Laurentiu Tatu

**Affiliations:** 1 Doctoral School of Biomedical Sciences, ‘Dunarea de Jos’ University, 800008 Galati, Romania; nechitaluiza2012@yahoo.com (L.N.); alexiaanastasia1998@yahoo.com (A.A.S.B.); 2 Department of Morphological and Functional Sciences, Faculty of Medicine and Pharmacy, “Dunărea de Jos” University, 800008 Galați, Romania; 3 ‘Sf. Apostol Andrei’ Clinical Emergency County Hospital, 800578 Galati, Romania; carinavoinescu@gmail.com (D.C.V.); corinampalivan@gmail.com (C.M.); 4 Clinical Medical Department, Faculty of Medicine and Pharmacy, ‘Dunarea de Jos’ University, 800008 Galati, Romania; lilibaroiu@yahoo.com (L.B.); nechitaaurel@yahoo.com (A.N.); camelia_busila@yahoo.com (C.B.); debita_mihaela@yahoo.com (M.D.); dralin_tatu@yahoo.com (A.L.T.); 5 ‘Sf. Cuv. Parascheva’ Clinical Hospital of Infectious Diseases, 800179 Galati, Romania; 6 ‘Sf. Ioan’ Clinical Hospital for Children, 800487 Galati, Romania

**Keywords:** acute myocardial infarction, COVID-19 prognosis

## Abstract

**Background/Objectives**: The newly emergent COVID-19 pandemic involved primarily the respiratory system and had also major cardiovascular system (CVS) implications, revealed by acute myocardial infarction (AMI), arrhythmias, myocardial injury, and thromboembolism. CVS involvement is done through main mechanisms—direct and indirect heart muscle injury, with high mortality rates, worse short-term outcomes, and severe complications. AMI is the echo of myocardial injury (revealed by increases in CK, CK-MB, and troponin serum markers—which are taken into consideration as possible COVID-19 risk stratification markers). When studying myocardial injury, physicians can make use of imaging studies, such as cardiac MRI, transthoracic (or transesophageal) echocardiography, coronary angiography, cardiac computed tomography, and nuclear imaging (which have been used in cases where angiography was not possible), or even endomyocardial biopsy (which is not always available or feasible). **Two-case-series presentations:** We present the cases of two COVID-19 positive male patients who were admitted into the Clinical Department of Cardiology in “Sfântul Apostol Andrei” Emergency Clinical Hospital of Galați (Romania), who presented with acute cardiac distress symptoms and have been diagnosed with ST elevation AMI. The patients were 82 and 57 years old, respectively, with moderate and severe forms of COVID-19, and were diagnosed with anteroseptal left ventricular AMI and extensive anterior transmural left ventricular AMI (with ventricular fibrillation at presentation), respectively. The first patient was a non-smoker and non-drinker with no associated comorbidities, and was later discharged, while the second one died due to AMI complications. **Conclusions:** From this two-case series, we extract the following: old age alone is not a significant risk factor for adverse outcomes in COVID-19-related CVS events, and that the cumulative effects of several patient-associated risk factors (be it either for severe forms of COVID-19 and/or acute cardiac injury) will most probably lead to poor patient prognosis (death). At the same time, serum cardiac enzymes, dynamic ECG changes, along with newly developed echocardiographic modifications are indicators for poor prognosis in acute cardiac injury in COVID-19 patients with acute myocardial injury, regardless of the presence of right ventricular dysfunction (due to pulmonary hypertension).

## 1. Introduction

As a newly emergent viral outbreak of the severe acute respiratory syndrome coronavirus-2 (SARS-CoV-2), manifested by the coronavirus disease 2019 (COVID-19), the disease came with a primary involvement of the respiratory system (ranging from asymptomatic disease forms to acute respiratory distress syndrome—ARDS) and also with major cardiovascular implications (acute myocardial infarction—AMI, arrythmias, myocardial injury, and pulmonary thromboembolism), be it either as direct complications of the disease or as exacerbations of a previous disease, as acute events or as post-acute sequelae [1,2]. Even from the early pandemic days, COVID-19 was found to be responsible for both arterial and venous thrombotic complications [3,4], with major echo in AMI patient medical care addressability, some studies reporting up to 50% fewer (or late) AMI patient admissions during the pandemic [5,6,7].

Acute myocardial injury is a relatively frequent event among patients suffering from COVID-19, correlating directly with the severity of this disease [1,2]. If patients suffering from coronary artery disease (as a long-term illness) or with atherosclerotic disease risk factors get infected with the SARS-CoV-2 virus, there is an increased risk of having an acute coronary syndrome (ACS) [1]. The risk of suffering from an AMI is reported as double in the first seven days after a patient is diagnosed with COVID-19, other studies reporting an AMI risk of up to five times higher for such patients (and even a tenfold risk of developing acute ischemic stroke) [3,8]. A possible mechanism for such major organ system involvement could be explained through a systemic inflammatory hyperreactivity, which is activated by the viral presence and mediated by various inflammatory circulating cytokines, determining atherosclerotic plaque instability with consequent rupture and AMI (with an incidence of up to 8.9% of COVID-19 patients) [1,8,9]. The cardiovascular consequences of COVID-19 can either manifest as an acute event, as stated earlier, or as long-term complications with severe sequelae [1,2,4].

## 2. Materials and Methods

We conducted searches in PubMed, Google Scholar, and Research Gate and examined meta-analyses and clinical trials from 2020 to 2024, using keywords such as acute myocardial infarction, treatment, therapy, clinical trials, and SARS-CoV-2. We searched for information about the emerging COVID-19 pandemic, in which effects appear first on the respiratory system, but also has severe effects on the cardiovascular system, manifesting as acute myocardial infarction, myocardial injury, arrhythmia, and pulmonary thromboembolism. At the same time, a two-case series is presented with the main objectives of paralleling the patients’ overall and general characteristics, COVID-19-related and cardiovascular symptoms, paraclinical aspects, treatments used, and outcomes, and to draw conclusions that will be compared to the literature data. We were particularly interested in the almost opposite patient characteristics and cardiovascular presentation and outcomes. In regards to the case series, the criteria for selection included: confirmed COVID-19 diagnosis through laboratory testing (PCR testing), confirmed AMI diagnosis (based on clinical presentation, EKG changes, and biomarker evidence), a temporal relationship to these two diseases (the onset of AMI symptoms occurred during active COVID-19 infection), severity of COVID-19 (mild to severe/critical, to explore the potential associations between COVID-19 severity and the incidence or outcomes of AMI), patient demographics (we have included patients with diverse demographic backgrounds in the research for potential disparities in the incidence and outcomes of such patients), comorbidity presence (and which types), clinical presentations of the AMI, and treatment modalities with possible impact on the patient outcomes. The exclusion criteria included other types of COVID-19 myocardial injury, such as myocarditis. Data collection and analysis was done with the help of the electronic and physical databases of the hospital, taking into consideration the abovementioned parameters. These rigorous criteria aid in enhancing the validity and generalizability of the research findings, in the hopes of shedding light on the COVID-19-AMI interplay, improving clinical practice, and public health strategies.

## 3. Results

### 3.1. COVID-19 Cardiovascular System Pathophysiology

SARS-CoV-2 is a virus with extensive organotropism, being able to inflict injury to various organ systems, such as respiratory (as main target), renal, or cardiovascular, and to different tissues (liver or brain). Its CVS involvement results in multiple possible complications, such as arrhythmias, myocarditis, myocardial injury, AMI, heart failure, or thromboembolic events [1,8,10].

There are three main mechanisms of CVS damage by the COVID-19 virus, each pathway leading to a type of injury:

- direct damage to the heart muscle by angiotensin-converting enzyme 2 (ACE-2) viral entry with inflammation and cell destruction.
- indirect damage owing to the ACE-2 downregulation after viral replication takes place, with consequent angiotensin II (Ang II)/angiotensin II receptor type I (AT1) system overactivation, with inflammatory, oxidative, and vasoconstrictive effects.
- indirect damage by B and T cell immune activation, with the development of a systemic inflammatory response and CVS stress (hypoxemic mechanism) [1,10,11,12].

As a relatively frequent event, myocardial injury is the result of cardiac muscle cell infection by the virus itself, with added effect, produced inflammation (with reactive oxygen species and free radicals’ production), microvascular thrombosis, and oxygen supply–demand imbalance [1,11,13]. This propensity toward heart muscle damage by the viral load is supported by genetic testing on autopsy heart samples, which identified the viral genome in almost half of cases. Associated changes in the heart muscle were found in the form of inflammation and fibrosis [1,11]. These changes were translated in increased values for CK and CK-MB, two indicators of myocardial damage [5].

Of particular interest is the interaction between the COVID-19 virus and the renin angiotensin system (RAS), through classic pathway activation, ACE-2 (found in the lung, myocardial pericytes, and circulatory endothelium) playing the role of RAS regulator. Through molecular studies, it was found that ACE-2 is the entry receptor of the SARS-CoV-2 inside the cell. This is achieved by activating the external membrane spike protein S through the transmembrane protease serine 2 (TMPRSS2). This entry receptor is afterwards internalized, SARS-CoV-2 releasing its RNA in order to replicate and initiate transcription of the virus’ genome, with the synthesis and assembly of viral structure proteins and the subsequent release of viral loads (exocytosis). After internal replication and exocytosis, the host cell can become disabled or even destroyed. Activation of Ang II/AT1 (patients having increased Ang II levels) leads to endothelial damage, inflammation, and vasoconstrictive and pro-coagulation effects, with vascular permeability increase and organ damage [1,8,14].

Regarding the indirect damage by B and T cell immune activation, there is the so-called ‘cytokine storm’, which was frequently observed in severe cases of SARS-CoV-2 infection (with severe lung and CVS disease), involving high serum levels of interleukins (IL-2, IL-7, IL-10, TNF-α) and finally leading to organ failure. Due to the high activated macrophage serum levels, other cytokines are being released (IL-6, IL1b). This systemic inflammatory response is responsible for heart cell apoptosis and/or fibrosis, inotropism, and hypercoagulability (due to procoagulant factor release) [1,8,13]. The endothelial dysfunction and microvascular lesions that occur are due to inflammation and adhesion molecule expression, along with the release of procoagulant factors, further accelerating the inflammatory and prothrombotic effects and interacting with atherosclerotic plaques, with rupture, thrombosis, and consequent AMI (a phenomenon that was also observed in influenza virus outbreaks) [1,11,13,14]. Inflammation is also responsible for the lack of equilibrium between oxygen supply and demand [1,11]. Endothelial cells are a preferentially targeted in COVID-19, and their morphological and functional damage in COVID-19 patients has a crucial effect, contributing to multiorgan dysfunction through hyperinflammation; this fact can be explained through a direct viral effect, oxidative stress, cytokine release, coagulation problems, and immune system response, but also a possible pre-existing endothelial dysfunction [15,16,17]. The effect is an acute endothelialitis/endotheliitis extending to the entire circulatory system, with abnormal vasoconstriction, inflammatory-cell-luminal plugging, intravascular thrombosis, affecting both arterial and venous circulations. Autopsy findings report finding apoptotic endothelial cells with viral inclusions, and microvascularization, with lymphocytic endothelitis (inflammation found around and infiltrating the blood vessels and endothelial cells). Apoptotic endothelial cells or dead endothelial cells were found in COVID-19 patients in the lung, heart, kidney, and small bowel. This virus-mediated apoptotic response disrupts the endothelial cell barrier and determines interstitial edema, circulating immune cell (that are activated) recruitment, along with the activation of platelets and the coagulation cascade [16].

As such, the CVS is targeted both directly (through viral damage to myocardial cells) and indirectly (through immune cell activation and ACE-2 downregulation), leading patients who suffer from CVS diseases or diabetes to having susceptibility to a more severe form of COVID-19 [1,18,19]. Patients with CVS involvement are reported as having high mortality rates, with worse short-term outcomes and severe complications [5,10,14].

### 3.2. AMI-COVID-19 Patient Characteristics

AMI is the main form of myocardial injury (revealed by increases in CK, CK-MB, and troponin serum markers—taken into consideration as possible COVID-19 risk stratification markers), with some subtypes being described in the literature:

- type 1—the result of coronary thrombosis, which develops in instances of plaque rupture—either by erosion or ulceration, or dissection.
- type 2—the result of mismatch between myocardial oxygen supply and demand (without thrombosis) [1,8,13].
- type 3—the result of an overlap between the first two subtypes [1].

COVID-19 patients are at risk for developing some form of myocardial injury, sometimes difficult to diagnose due to atypical presentation (chest pain absence). From these, those with AMI (with or without prior CVS disease) were reported to have higher mortality rates, arrhythmias, acute respiratory distress, acute kidney injury, and electrolyte alteration [1]. Such patients were the unfortunate ones, due to major drawbacks in AMI-COVID-19 patient care: late presentation to the emergency unit (due to possible fear of contamination), prolonged door to balloon time, worsening outcomes, and decrease in ST-elevated AMI activations [20,21,22].

### 3.3. AMI-COVID-19 Imaging Studies

When studying myocardial injury, physicians can make use of imaging studies such as cardiac MRI, transthoracic (or transesophageal) echocardiography, cardiac computed tomography, and nuclear imaging (which have been used in cases where angiography was not possible), or even endocardial biopsy (which is not always available or feasible) [1,12,23]. In COVID-19 times, medical personnel were advised to limit patient contact and aerosol-generating procedures (such as transesophageal echocardiography) [23].

Echocardiography was initially (and as first option) used in monitoring COVID-19 patients with or without cardiac injury, being easy to use, available, and portable [12,24]. Echocardiographic changes seen in a COVID-19 patient can be dilatation and dysfunction of the right ventricle (especially in critical patients, with tricuspid annular plane systolic excursion (TAPSE) reduction, a mortality predictor), wall thickening, dyskinesia (regional or global), with decreased left-ventricle ejection fraction [1,12,25,26].

CMR (cardiac MRI) is the most important tool when characterizing myocardial injury (especially myocarditis), complementing echocardiographic findings, most importantly when a non-ischemic type of injury is suspected (and in an ambulatory setting) [12,24]. The literature data reports up to 78% of patients with evidence of myocardial injury—edema, inflammation, necrosis, and late lesions (myocarditis-like scar, ischemia, and infarction) [1,23]. This particular type of imaging investigation was also used in order to compare cardiac injury cases in COVID-19 patients and those developed after the vaccine [27].

Cardiac CT was used in COVID-19 patients for evaluating lung disease, but in cases with cardiac injury, by using contrast CT, physicians could evaluate a delayed subendocardial enhancement (a marker for myocarditis); it was also used in the evaluation of coronary artery disease in such patients [12,24]. Fluorodeoxyglucose (FDG) positron emission tomography (PET) was also used in COVID-19 cases with myocardial injury and was able to identify inflammation and thus, myocarditis, (revealed by a segmental FDG uptake and matching defects in perfusion), but it is not recommended as a routine practice [12,24].

Histopathology data revealed findings such as inflammation, microvascular thrombi, and intraluminal megakaryocytes [1].

### 3.4. Two-Case Presentation Series

In March 2022, two COVID-19 positive male patients were admitted into the Cardiology Clinic of the “Sfântul Apostol Andrei” Emergency Clinical Hospital of Galați suffering from acute cardiac distress symptoms, and werediagnosed with ST elevation AMI.

**Figure 1:**
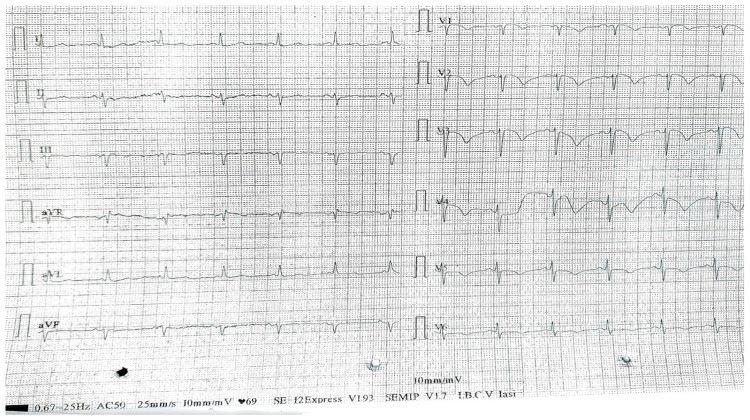
ECG findings—sinus rhythm, with 75 bmp, left anterior bundle branch block, QRS complex duration of 80 s, slow progressing R waves in V1 to V4, and negative T waves in V1–V6.

The first patient was an 82-year-old non-smoking male with no alcohol consumption, or prior heart disease history, presenting with prolonged retrosternal chest pain (angina) extending in both arms. The patient was diagnosed with anteroseptal AMI and was found to be positive for COVID-19, with no other sick contacts. On examination, the patient had a body temperature of 36.4 Celsius, with a heart rate of 62 bpm (beats per minute), a blood pressure of 116/75 mmHg, and an oxygen saturation of 97% without additional oxygen therapy. The physical examination revealed no significant changes, while the laboratory results revealed a non-specific inflammatory syndrome (an ESR of 65 mm/h, a fibrinogen level of 828 mg/dL, an LDH of 637 U/L, and a C reactive protein of 69.1 mg/L) with cardiac cytolysis syndrome (CK-MB—109 U/L) and a modified ECG with a 1–2 mm elevation of the ST segment in V1 to V3 derivations with negative T waves from V1 to V6 (Figure 1). On echocardiography, an enlarged heart with an akinetic region of the left ventricle was found (anterior wall and septum) having a markedly decreased left-ventricle ejection fraction (LVEF) of only 10%, with right heart dysfunction and pulmonary hypertension; mitral and tricuspid valve regurgitation was also found. The coronary angiography report revealed a left main artery atherosclerotic stenosis of 40%, with a left anterior descending artery having acute thrombotic occlusion and a left circumflex artery with 80% stenosis, and angioplasty was attempted without success. A chest X-ray was done, reporting left basal lung changes with alveolar condensation.

During the patient’s hospital stay, his general health improved under strict treatment for the coronary disease (dual antiplatelet treatment, betablockers, nitrate derivates, statins, aldosterone antagonists, and factor Xa inhibitor), with antiviral and antibiotic treatment for his moderate COVID-19 disease (Favipiravir, cephalosporin—ceftriaxone). The patient was discharged without angina symptoms, with a corrected lipid profile but with an increased NT proBNP serum level (4133 pg/mL).

**Figure 2:**
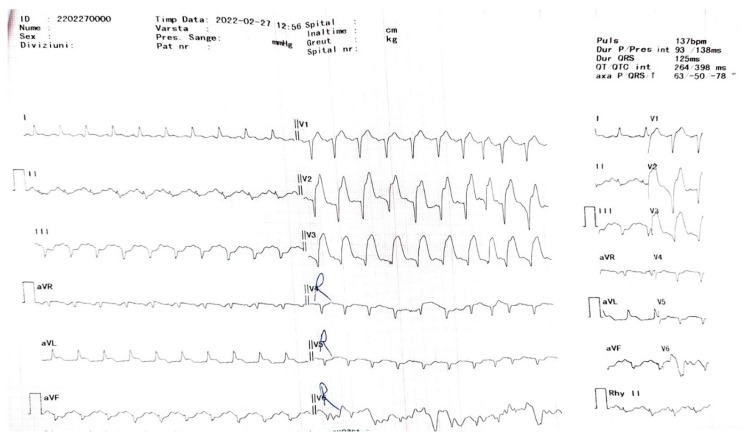
ECG findings—sinus rhythm with 120 bpm, PR interval of 120 ms, ORS interval of 80 s, ST elevation of 5 mm in V2 to V6, DI, aVL, and Q wave present in V2-V6, DII, DIII, and aVF.

**Figure 3:**
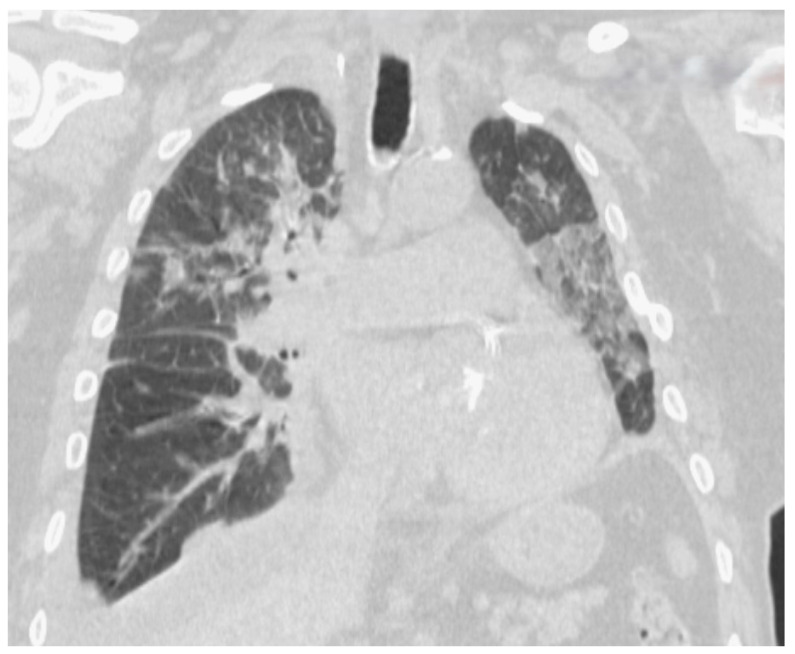
Chest CT—diffuse left and right superior lobe alveolar ground-glass opacities with crazy-paving pattern and bilateral pleural effusion.

The second cardiac patient was a 57-year-old chronic alcohol consumer and smoker, with a complex history involving ischemic stroke, hypertension, chronic alcoholic hepatitis, and high serum cholesterol was admitted in GCS = 3 points coma with a severely affected health state after resuscitated cardiac shock, which developed after initially intense central chest pain. The SARS-CoV-2-positive patient (known to our clinic) was suffering from an extensive anterior transmural AMI, complicated with primary ventricular fibrillation and cardiogenic shock. On examination, the patient had a body temperature of 37 Celsius, with a heart rate of 146 bpm, blood pressure of 108/58 mmHg, with intravenous dobutamine and an oxygen saturation of 100% on mechanical ventilator machine. The physical examination revealed equally poorly reactive and constrictive pupils, facial asymmetry, pachyonychia, pale skin with lower extremity cold, marbled skin (livedo reticularis), with perspiration, cachexia, and unstable hemodynamics. The laboratory results revealed a non-specific inflammatory syndrome (fibrinogen—733.60 mg/dL, LDH—983 U/L, and C reactive protein—218.40 mg/L) with cardiac cytolysis syndrome (CK-MB—55.70 ng/dL, myoglobin > 500 ng/mL, and troponin I > 20 ng/mL). The ECG evaluation revealed a 5 mm elevated ST segment elevation in V2 to V6, DI, aVL, with Q waves present in V2–V6, DII, DII, aVF, 80 s QRS complex (Figure 2). On transthoracic echocardiography, the left ventricle was dilated with extensive akinesia of the ½ apical anterior wall and 2/3 apical septum, with an LVEF of 30%, with no right cavity changes or pulmonary hypertension. A chest X-ray was done, reporting bilateral alveolar condensates. The chest CT COVID-related lung changes (Figure 3) were found with diffuse left and right superior lobe alveolar ground-glass opacities with crazy-paving pattern and bilateral pleural effusion.

During the patient’s hospital stay, his general health declined in spite of the administered treatment for the coronary disease (dual antiplatelet treatment, anticoagulants, diuretics, and aldosterone antagonists, along with dexamethasone, dobutamine), with antiviral and antibiotic treatment for the severe COVID-19 disease (Remdesivir, fluoroquinolone—levofloxacin), and other symptom-related and sustaining treatment (acetylcysteine, proton pump inhibitor, B1, B6, C and D vitamins, zinc, metamizole). The patient died after four days of in-hospital admission in the ICU, succumbing to unresponsive cardiac arrest.

## 4. Discussion

The emergence of the novel SARS-CoV-2 virus with the widespread global pandemic of COVID-19 translated in an unprecedented health crisis, affecting patients and health departments across multiple countries [28,29]. A system frequently and severely affected by this disease is the cardiovascular one, although the upper and lower respiratory ones are the main targets, with symptoms raging from mild (cough, shortness of breath) to severe (dyspnea, respiratory failure) [30,31]. COVID-19 patients suffering from pre-existing cardiovascular pathology (most frequentlyhypertension—59.8%) were found to have poor outcomes, with increased mortality rates (associating severe COVID-19 forms and frequent ICU admissions) due to (acute and chronic) cardiovascular injuries, such as arrhythmias, myocarditis, infarction, endothelial cell injury, myocardial interstitial fibrosis, venous, and arterial thrombo-embolic events, heart failure, cardiogenic shock, right ventricular dysfunction, Takotsubo cardiomyopathy, and pericardial effusion [28,30,32,33,34,35]. The patient from the second case presentation had a poor outcome (death), associating a severe COVID-19 form with previous cardiovascular pathology, hypertension, supporting the current medical literature reports.

Various mechanisms are considered as being involved in the pathogenesis of this complex disease: cytokine storm, direct myocardial cell injury (through the ACE2 receptors), catecholamine surge, electrolyte imbalance, systemic inflammation, and hypoxia [30,33,36]. Acute cardiac injury (defined as increased cardiac biomarker levels and/or newly developed ECG or echocardiographic abnormalities) was found to be the most prevalent complication of cardiovascular system involvement by COVID-19, affecting elderly patients with comorbidities and poor outcomes; as such, this type of cardiac injury is considered an independent risk factor for the severe COVID-19 forms and also an independent predictor of patient mortality [30,37,38,39]. Arrythmias were found in up to 60% of cases, possibly being responsible for the hemodynamic instability and overall complications, with an underlying mechanism determined by direct or indirect endomyocardial injury [30,40,41]. Other possible risk factors for cardiovascular events in COVID-19 (but also for severe forms of COVID-19 and higher mortality risk) are considered—diabetes, hypertension, previous stroke or cardiovascular events, COPD and other respiratory diseases, chronic kidney or liver disease, and malignancy [30,37,42,43].

As the most frequent COVID-19-related CVS complication, acute cardiac injury was found in almost 25.9% of cases, manifesting with higher frequency as acute coronary syndrome, cardiogenic shock, or heart failure [30,44,45].

The two-case series reported in the current work revealed two almost entirely different clinical, evolutionary, and paraclinical aspects of the COVID-19 patients. Although both were male patients, the age difference is significant, along with personal history and initial hospital admission clinical manifestations. The first SARS-CoV-2 positive patient, an 82-year-old with no unhealthy or risky behaviors, no associated diseases (zero comorbidities), was at the first in-hospital admission for an anteroseptal AMI with wall akinesia, right heart dysfunction, and pulmonary hypertension, with a moderate form of COVID-19. The second patient, a much younger 57-year-old man, a chronic alcohol abuser and smoker, with prior hospital admissions for important associated pathologies—ischemic stroke, hypertension, chronic hepatitis, and hypercholesterolemia, was currently admitted for an extensive anterior transmural AMI with ventricular fibrillation, cardiogenic shock, and a coma of 3 point on the GCS. The first patient survived, being discharged with an improved general health state, while the second one died, presenting with reported medical literature, poor outcome predictors (severe COVID-19, prior hypertension, and other comorbidities—stroke, serum cardiac enzymes, ECG, and echocardiography changes).

Regarding the poor outcome, risk factors related to COVID-19 and acute cardiac injury (from which both patients suffered, confirmed by cardiac enzyme assay, ECG and echocardiography), our patients presented the following:

- the first patient (associating right ventricular dysfunction)—had only one COVID-19-associated CVS events risk factor—old age;
- the second patient—suffered from severe COVID-19 (associated with hypertension as a risk factor for severe disease and associated CVS events), had significant comorbidities (previous stroke and chronic liver disease) as risk factors for CVS events in COVID-19; this patient developed arrhythmia (ventricular fibrillation) with hemodynamic instability and death;

Two rather straightforward conclusions can be drawn from the two latter statements—old age alone is not a significant risk factor for adverse outcomes in COVID-19-related CVS events, and that the cumulative effects of several associated risk factors (be it either for severe forms of COVID-19 and/or acute cardiac injury) will most probably lead to poor patient prognosis (death). At the same time, serum cardiac enzymes and ECG changes, along with newly developed echocardiographic modifications, are indicators for poor prognosis in acute cardiac injury in COVID-19 patients, regardless of right ventricular dysfunction (with pulmonary hypertension).

Old age is not only a number but a state of health, which poses the patient at risk not only for an easier SARS-CoV-2 infection, but also for developing severe forms of COVID-19 (with or without CVS abnormalities), with higher mortality rates [46,47]. As such, associated with old age, are: increased ACE2 receptor expression, immune dysregulation (resulting also from sex steroids and growth hormone decrease), gut microbiota changes (contributing to the cytokine storm), mitochondrial dysfunction and oxidative stress in immune cells and pneumocytes—associated with senescence, life-style associated factors (compromised physical activity, nutrition) and aging-associated illnesses (especially CVS, diabetes mellitus) [46]. Multiple 2020 studies (Guo et al., Song et al., Wang et al., Zhang et al., Wei et al., Lian et al., Godaert et al.) reported CVS events in COVID-19 patients in the form of acute cardiac injury in up to 21.6% of them. Elderly patients suffered from frequent arrhythmias (up to 14.81% and, in non-survivors, 20.6%) and cardiac failure/insufficiency (up to 22.22% and, in non-survivors, 42.4%) [48,49,50,51,52,53,54]. Our finding that old age alone is not in fact a stand-alone risk factor regarding COVID-19 and its complications (including mortality) is supported by the current literature, which reports a relatively weak influence (after the adjustment for age-related risk factors—mostly comorbidities—diabetes, CVS, hypertension, stroke, respiratory and renal disease, and, respectively, immune dysregulation, which is involved also in tumor development and progression) [55,56].

Up to 90% of COVID-19 patients revealed ECG changes due to various causes—myocardial injury, microthrombi, plaque rupture, hypoxic injury, and cytokine storm, with various arrhythmias or ST and T wave alterations. Poor outcome is associated with QT interval prolongation, T wave and ST segment modifications, and ventricular fibrillation or tachycardia [57]—findings that were revealed in the case of the second patient, with death as the final complication and poor outcome. We come in support of the current medical literature, which states that ECG on admission for COVID-19 patients can be used as a mortality risk predictor [58]. Echocardiographic studies in COVID-19 patients revealed that severe forms are associated with right and/or left ventricular dysfunction (±pericardial effusion) with wall dyskinesia (hypokinesia/akinesia), another tool which can be used for prognosis evaluation; these changes were also associated with cardiac enzyme elevations; some studies imply the use of procalcitonin as a predictive factor for COVID-19 evolution [59,60,61].

## 5. Conclusions

COVID-19 is a multi-system disease that affects mainly the respiratory one, with important implications regarding CVS disease. From the two COVID-19 patients with AMI presented in the current work, the following was reported: old age alone is not a significant risk factor for adverse outcomes in COVID-19-related CVS events, and that cumulative effects of several associated risk factors (be it either for severe forms of COVID-19 and/or acute cardiac injury) will most probably lead to poor patient prognosis (death). At the same time, serum cardiac enzyme and ECG changes, along with the newly developed echocardiographic modifications, are indicators for poor prognosis in acute cardiac injury in COVID-19 patients, regardless of right ventricular dysfunction (with pulmonary hypertension).

## Data Availability

Data Availability Statements are available on request, through the corresponding author.
